# Duck Hepatitis B Virus cccDNA Amplification Efficiency in Natural Infection Is Regulated by Virus Secretion Efficiency

**DOI:** 10.1371/journal.pone.0145465

**Published:** 2015-12-29

**Authors:** Yong-Yuan Zhang

**Affiliations:** HBVtech, Germantown, Maryland, United States of America; National Institute of Health - National Cancer Institute, UNITED STATES

## Abstract

Previous mutation based studies showed that ablating synthesis of viral envelope proteins led to elevated hepadnaviral covalently closed circular DNA (cccDNA) amplification, but it remains unknown how cccDNA amplification is regulated in natural hepadnaviral infection because of a lack of research system. In this study we report a simple procedure to prepare two identical duck hepatitis B virus inocula, but they possess 10-100-fold difference in cccDNA amplification in infected cell culture. We demonstrate that the infected cells with higher cccDNA amplification significantly reduce the virus secretion efficiency that results in higher accumulation of relaxed circular DNA (rcDNA) and DHBsAg in the cells. The infected cells with lower cccDNA amplification significantly increase the virus secretion efficiency that leads to lower intracellular rcDNA and DHBsAg accumulation. In contrast with the findings generated in the mutation based experimental system, the regulation of cccDNA amplification in natural hepadnaviral infection bypasses direct regulation of the cellular envelope proteins concentration, instead it modulates virus secretion efficiency that ultimately impacts the intracellular rcDNA concentration, an important factor determining the destination of the synthesized rcDNA in infected cells.

## Introduction

Hepadnaviruses are a family of hepatotropic DNA viruses and the family members include human hepatitis B virus (HBV), woodchuck hepatitis virus (WHV) and duck hepatitis B virus (DHBV)[1]. Hepadnaviruses share similar genetic organization, which mainly consists of three open reading frames that encode viral envelope, pre-core/core, and polymerase (P) proteins [2]. Hepadnaviral lifecycle starts with virus entry through binding to cellular receptors. An identified HBV receptor is sodium taurocholate cotransporting polypeptide (NTCP)[3]. The virus, once entered into the cell, is uncoated, and the viral nucleocapsid is transported to the nucleus where the viral genome is released. The released viral genome, which is partially double stranded and can’t directly function as a template for RNA transcription. In the nucleus it is converted to covalently closed circular DNA (cccDNA) [4–6]. Three main viral RNA species including pregenomic RNA and two *S* RNAs are transcribed from the cccDNA and migrated to the cytoplasm.

Viral proteins including polymerase, core and envelope proteins are translated using the viral RNAs templates. Then the pregenomic RNA is interacted with the polymerase protein[7] and is reverse transcribed, resulting in synthesis of the minus strand DNA in the nucleocapsid [8]. Often, an incomplete plus strand DNA is synthesized using the minus strand DNA template [9, 10]. The mature nucleocapsid that carries the newly synthesized relaxed circular DNA (rcDNA) can be used for assembling virions that can be secreted to outside cell, or can be transported to the nucleus for replenishing cccDNA pool (intracellular pathway)[11]. Thus some newly synthesized viral rcDNA molecules serve as source for producing virions while other for amplifying cccDNA pool. The factors determining the cellular rcDNA molecule flow direction remains much unknown.

The cccDNA is required for establishing and maintaining HBV infection [12]. Current antiviral treatment can potently inhibit HBV replication, but rarely clears chronic HBV infection because of persistence of cccDNA [13]. Thus understanding of regulating mechanisms for cccDNA amplification is of great importance in both hepadnaviral biology and new antiviral drug development. DHBV and DHBV infected cell culture are valid and convenient models to address this important issue [1, 14]. In identifying viral factors that participate in regulating cccDNA amplification, it was found that ablation of the viral pre-S/S protein synthesis resulted in increased cccDNA amplification in DHBV system [12, 15–17]. Later, the envelope proteins were also found to participate in inhibiting cccDNA amplification in HBV system [18–20]. An inverse relationship between cccDNA and envelope proteins levels was observed [18]. It is proposed that the regulation of cccDNA amplification by the envelope proteins is mediated through interaction between the envelope proteins and nucleocapsid [12].

In natural HBV infection the amount of hepatitis B surface antigen (HBsAg) is overwhelmingly produced and maintained [21]. The HBsAg mainly consists of the small S protein, but it also carries both M and L proteins [22], implying that a high HBsAg level is accompanied by correspondingly high levels of the M (pre-S2) and L (pre-S1) proteins. Thus the high level of envelope proteins likely promotes active interaction with the capsid during natural hepadnaviral infection. There might even be a possibility that the nucleocapsids can be preferably utilized for assembling virions because the capsids are outnumbered by the envelope proteins. Indeed it was observed that co-expression of envelope proteins limited the completion of plus-strand DNA synthesis [18], implying the mature nucleocapsid is in short supply to meet the demand of ongoing interaction between the envelope proteins and the capsid, which could lead to fewer rcDNA molecules available for replenishing cccDNA pool. Based on such reasoning, an inefficient cccDNA amplification or a steadily low cccDNA level in natural HBV infection can be expected unless there is a main regulation independent from modulating the cellular envelope proteins concentration. Varied and higher copies of cccDNA per cell were indeed detected in chronic HBV, DHBV and WHV infection [4, 23–25]. The cccDNA amplification kinetics in single individual cells isolated from 6 sequential liver biopsies taken from a chronically infected duck was found to be very dynamic [26], suggesting an effective regulating system for cccDNA amplification in natural hepadnaviral infection.

We wonder how the regulation of cccDNA amplification is accomplished in natural infection in which individual envelope proteins are not ablated and HBsAg remains high even after HBeAg seroconversion [27, 28]. Mutation based experimental system that ablates expression of individual envelope proteins can’t effectively address this issue. Ideally, a system that resembles natural infection, and keeps the experimental manipulation at minimum, is preferred. For instance, the same primary cell culture that is separately infected with two identical inocula, but with significant difference in cccDNA amplification would be a good system for analyzing how the difference in cccDNA amplification is generated. However, such experiment system can’t be easily established. Lack of such system has blocked the progress in studying regulation of cccDNA amplification in natural hepadnaviral infection.

In this study, we were able to investigate the cccDNA amplification in DHBV (WT) infected cell culture that was inoculated with two identical DHBV inocula, but with 10-100-fold difference in amplifying cccDNA. We demonstrate that higher level cccDNA amplification is accompanied by reduced virion secretion and elevated intracellular rcDNA level while lower level of cccDNA amplification is associated with efficient virion secretion and lower level of intracellular rcDNA level.

## Materials and Methods

### Animal experiment

The animal experiment in this study was carried out in strict accordance with the recommendations in the Guide for the Care and Use of Laboratory Animals of National Institutes of Health. The protocol for the proposed duck experiment that was used preparation of primary duck hepatocyte (PDH) culture, was approved by Institutional Animal Care and Use Committee at University of New Mexico School of Medicine (permit number: 0352). For each experiment, 10 female one-day-old Peking ducklings were purchased from Metzer Farms (Redlands, Calif.). All ducklings were comfortably housed in the animal research facility (ARF) of University of New Mexico School medicine. The ARF is staffed with a board certified veterinarian with Doctor Degree of Veterinarian Medicine and 8 laboratory animal technicians (LAT). The LAT staffs are daily on site, and the veterinarian is available at all times. The ARF is maintained as a specific pathogen free facility. The ducks were daily feed with wet mash containing 20% proteins and subjected to no water restriction. The LAT staffs daily conduct routine husbandry procedures (e.g., cage cleaning, feeding, and watering) and check animals to assess their condition. In addition, all animals were daily weighed and documented by the author and his assistant to monitor the growth rate and general well-being. If animals exhibited any indication of bacterial infection or distress, the LAT staff conferred with us to recommend appropriate antibiotics, analgesics, or other pharmaceuticals. The veterinary staff intervened with euthanasia if an animal was severely ill or suffered grave distress, for instance if it was no longer able to stand up, or walk or eat/drink or breathe or it suffered bleeding or progressively lost the weight. There was no single incidence that a duck died from severe illness or distress. All ducks were euthanized at the end of experiment (the endpoint was the success of PDH that took approximately two weeks) by IV injection of 0.5 ml of phenobarbital (30 mg/ml) consistent with recommendations of AVMA Guidelines on euthanasia. The quick action of phenobarbital minimized animal distress.

### Primary Duck hepatocyte culture (PDH)

Primary duck hepatocyte culture was either prepared in the lab following the protocol previously described [29], or purchased from Scikon Inc (Research Triangle Park, NC). Cultures were seeded at 1E6 cells per 60 mm dish (Falcon) or 5E5 cells per well in 6-well plate (BD) in L-15 medium (Gibco) The medium was changed and collected daily from day 4 to day 8 after the inoculum was replaced at day 1 post inoculation. All cells were harvested at day 8.

### Inoculum

One-day-old Peking ducklings were purchased from Metzer Farms (Redlands, Calif.). Congenitally DHBV-infected ducklings were detected after screening by dot blotting for viral DNA [30]. Serum samples were pooled from daily bleedings between day 3 and 10. The pooled serum samples were aliquoted and stored at -20 C. Aliquots of stored serum were later removed from the freezer and stored at 4 C for either 4 or 8 weeks before use for inoculation. Normal duck serum samples from uninfected animals were collected and stored at -20 C and used as controls.

### Isolation of replicative intermediates and cccDNA from cells

Cells were lysed with 1% NP40 in 50:1 TE buffer, pH 7.5. Extraction of replicative intermediates from the supernatant follows the protocol developed by the Summers’ laboratory[16]. The pellet portion was used for isolation of cccDNA after alkaline denaturation, potassium acetate precipitation, and phenol extraction at pH 5 [31]. We followed the published procedure [4] for southern blotting and hybridization except that the DNA probe was replaced with a full length riboprobe, which hybridizes the minus stranded viral DNA.

### Determining copy numbers of cccDNA and replicative intermediates by qPCR

Purified cccDNA was subjected to EcoRI digestion before PCR amplification. Amplification of cccDNA was conducted with a pair of primers that flank the cohesive region (DHBV 2407 [5_ TGT CCC GAG CAA ATA TAA TCC] and DHBV 2840R [5_ TGT GTA GTC TGC CAG AAG TCTTC]). The specificity for amplification of cccDNA was tested by comparing EcoRI-cut templates with uncut templates. The detection of cccDNA was increased by 40- to 120-fold by EcoRI cleavage compared with uncut template (data not shown). Because only linearized cccDNA can be efficiently denatured by heat to allow PCR amplification, this result suggested that the overwhelming majority of templates amplified by this primer set after digestion were derived from cccDNA.

For real-time PCR analysis of copy number of cccDNA and replicative intermediates, 5μl extracted replicative intermediates or EcoRI-cleaved cccDNA was mixed with 10 μl 2x SYBR green supermix (Bio-Rad), primers, and water in a total volume of 20 μl. Primer DHBV2548 [5_ TTC GGA GCT GCT TGC CAA GGT ATC] and DHBV2840R were used for detection of replicative intermediates. The qPCR was carried out in iCycler (Bio-Rad) or ABI 9700 HT instrument. The specificity of PCR products was verified by melting curves. In order to have accurate standard plasmid controls, the supercoiled plasmid containing the DHBV genome was isolated from an agarose gel and recovered using a commercial gel purification column (QIAGEN). The optical density at 260 nm was measured to determine the copy number, and the purified plasmid was linearized by EcoRI cleavage.

### ELISA detection of Duck Hepatitis B surface antigen (DHBsAg) in both medium and cell lysate

The detection procedure followed a published protocol [32]. In brief, duplicate wells of a 96-well microtiter plate (Nunc ELISA plate) were coated with 100 μl of a 1/100 dilution of serum drawn from the 3-day old duckling that was known to contain high titer of maternal neutralizing antibody [33] and incubated at 4 C overnight. Medium (diluted 1/10 with PBS) or cell lysate (diluted 1/100 with PBS) were added to the wells in a 100 μL volume. The monoclonal anti-DHBsAg (7C.12 that detects both DHBV pre-S and S proteins [34], a gift from Dr. JT Guo, Drexel University) was added at 1/5000 dilution. Finally the wells were incubated with horseradish peroxidase (HRP)-conjugated rabbit anti-mouse polyclonal antibodies (Life Technologies) at a 1/1000 dilution. The plate was read at 490 nm. A high-titer DHBsAg-positive serum and the medium from uninfected culture were included as positive and negative controls. The relative amount of DHBsAg was calculated as follows: Mean optical density (OD) value from duplicate wells were multiplied by the dilution factor and the total volume of medium or cell lysate.

### Antibody capture of nucleocapsids and virions retained in the cell lysate

Briefly, 96-well microtiter plates (Nunc ELISA plate) were coated in duplicate with 100 μl of either rabbit anti-DHBV core antibody (a gift of Dr. Summers, University of New Mexico) or mouse anti-DHBsAg (7C.12), each of which was 1/100 diluted in 0.1M sodium bicarbonate buffer pH 9.6 and adsorbed at RT for 2 hours. The coated plated was washed, then 100 μl cell lysate diluted at 1/100 with 10:1 TE buffer, pH 7.5, was added and the plate incubated for 1h at RT. The plate was washed and bound virions and nucleocapsids were subjected to proteinase K digestion (100 μl at 1mg/ml) at 55 C for 1 hr to release viral DNA, then The Proteinase K was inactivated at 85 C for 15 min. The released viral DNA was ready for qPCR. DHBV DNA copies per captured portion was determined through qPCR of serial dilutions. The reagents and procedure for qPCR were the same as above described for rcDNA detection. The ratio of DHBV DNA copies in the captured nucleocapsids and the captured virions was calculated.

### Determination of virus entry efficiency

We followed the published method [35]. Briefly, the primary duck hepatocyte culture was inoculated with the DHBV positive serum at MOI 40:1 for 4 hours. Next the inoculum was removed and washed with PBS for 3 times. Then the cells were treated with 0.5 ml of 0.25% trypsin-EDTA solution per dish for 15 min at 37°C. The trypsin was inactivated by adding 0.5 ml medium with 10% FBS. The detached cells were transferred to Eppendorf tubes, pelleted and washed by centrifugation before being suspended and lysed for DNA extraction.

### Statistical analysis

Variables were summarized in the form of means and standard deviations where they were applicable. Means were compared by two-sided Student’s t test. A *p* value of ≤0.05 was considered statistically significant.

## Results

### Prior storage of serum samples at 4 C for an extended period of time reduced accumulation of intracellular replicative intermediates

We frequently used the congenital DHBV positive sera as inocula for infecting primary duck hepatocyte (PDH) culture and animals. The DHBV genotype in our congenital DHBV positive sera is DHBV16 [36] based on our sequence analysis (data not shown). The congenital DHBV positive serum inoculation is generally referred as wildtype (WT) infection as opposed to the mutant viruses that are experimentally engineered [37].

**Fig 1:**
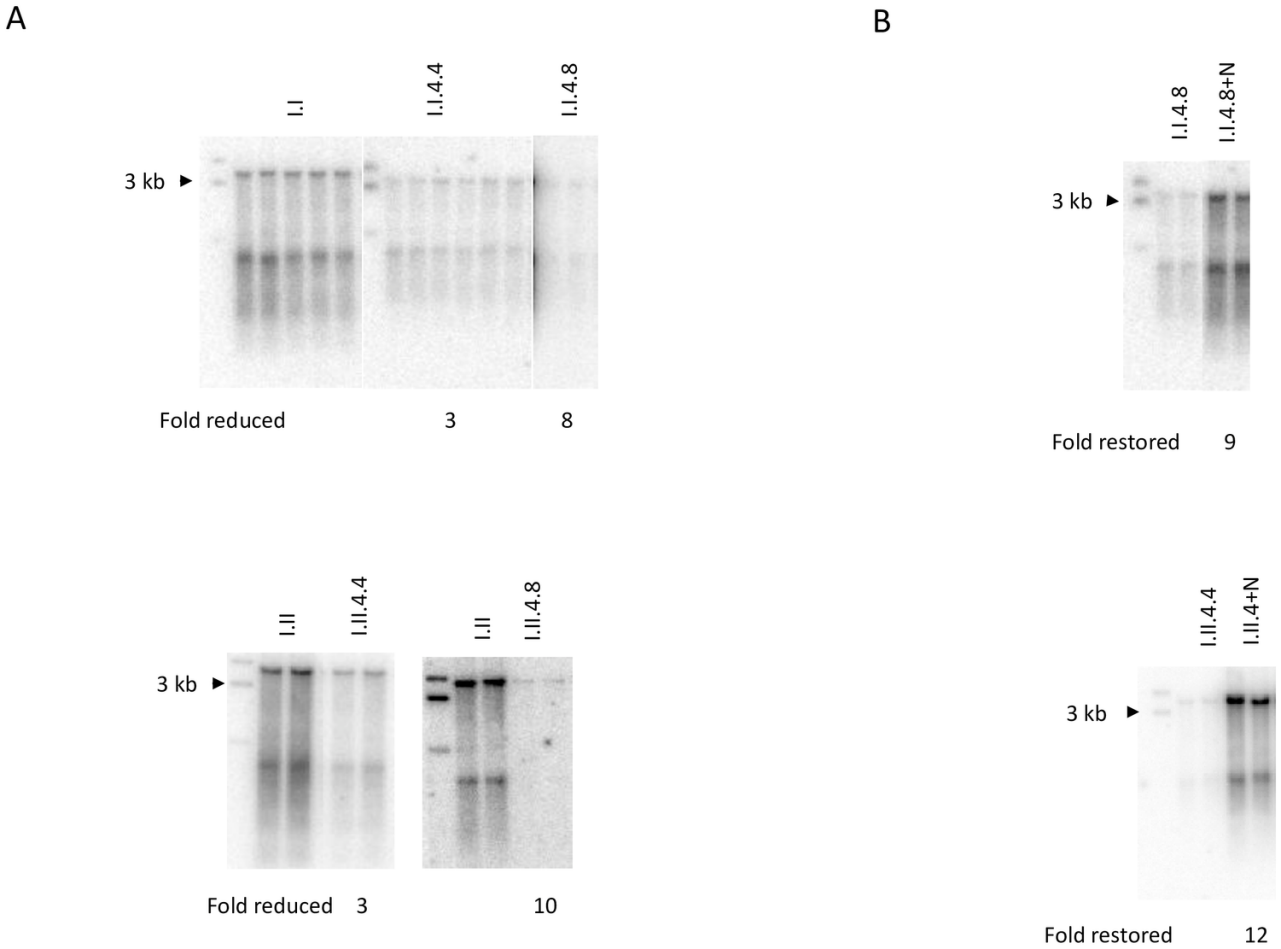
Storing serum samples at 4 C for an extended period of time reduced accumulation of intracellular replicative intermediates (harvested at day 8 pi). **A.** progressively reduced accumulation of intracellular replicative intermediates was observed over extending 4 C storage of the same aliquot inocula. **B.** upon the inoculation, an addition of 150 μl normal duck serum to the cells infected with the inoculum stored at 4 C for 8 weeks, restored the accumulation of intracellular replication intermediates in the infected cells. One of 6 representative experiments is shown. All samples were tested in duplicate or more replicate. I.I and I.II: Inoculum I and II stored at 20 C; I.I.4.4 and I.I.4.8, I.II.4.4 and I.II.4.8: the same two inoculum aliquots stored at 4 C for 4 and 8 weeks, respectively; +N. addition of 150 μl normal duck serum upon inoculation.

Sometimes, we stored the remaining serum aliquots at 4 C. We noticed that when sera that had been stored at 4 C for a few weeks (treated serum) was used as inoculum a reduced accumulation of intracellular replicative intermediates in the infected cells, compared with serum inoculum stored at -20 C (standard serum). We also noted that the degree of reduced accumulation of intracellular replicative intermediates was dependent on the length of time the serum was stored at 4 C (Fig 1A).

We wondered why and reasoned that the damage either to the virus or to serum proteins that may regulate the efficiency of accumulating replicative intermediates was responsible for the difference between treated and standard serum inocula. To distinguish between these two scenarios a small amount of normal (DHBV negative) duck serum was added to the treated serum inoculated cells. The results showed that the accumulation of replicative intermediates in the cells infected with the treated inoculum after adding 150 μl normal duck serum was restored to a comparable level as the standard inoculum stored at -20 C (Fig 1B). The results were highly reproducible after 6 rounds of repeated experiments with inoculum I and II. We concluded, that the storage at 4 C does not substantially damage the virus, but instead degrades a labile serum component(s) that regulates the accumulation of replicative intermediates in the infected cells. We tested more than a dozen of different DHBV serum inocula that were stored at 4 C for extended periods (data not shown), and we observed the same pattern as shown in Fig 1.

### The virus entry in the treated inoculum remained efficient, and the reduced accumulation of intracellular replicative intermediates was accompanied by the reduced cccDNA amplification in the cells infected with the treated inoculum

**Fig 2:**
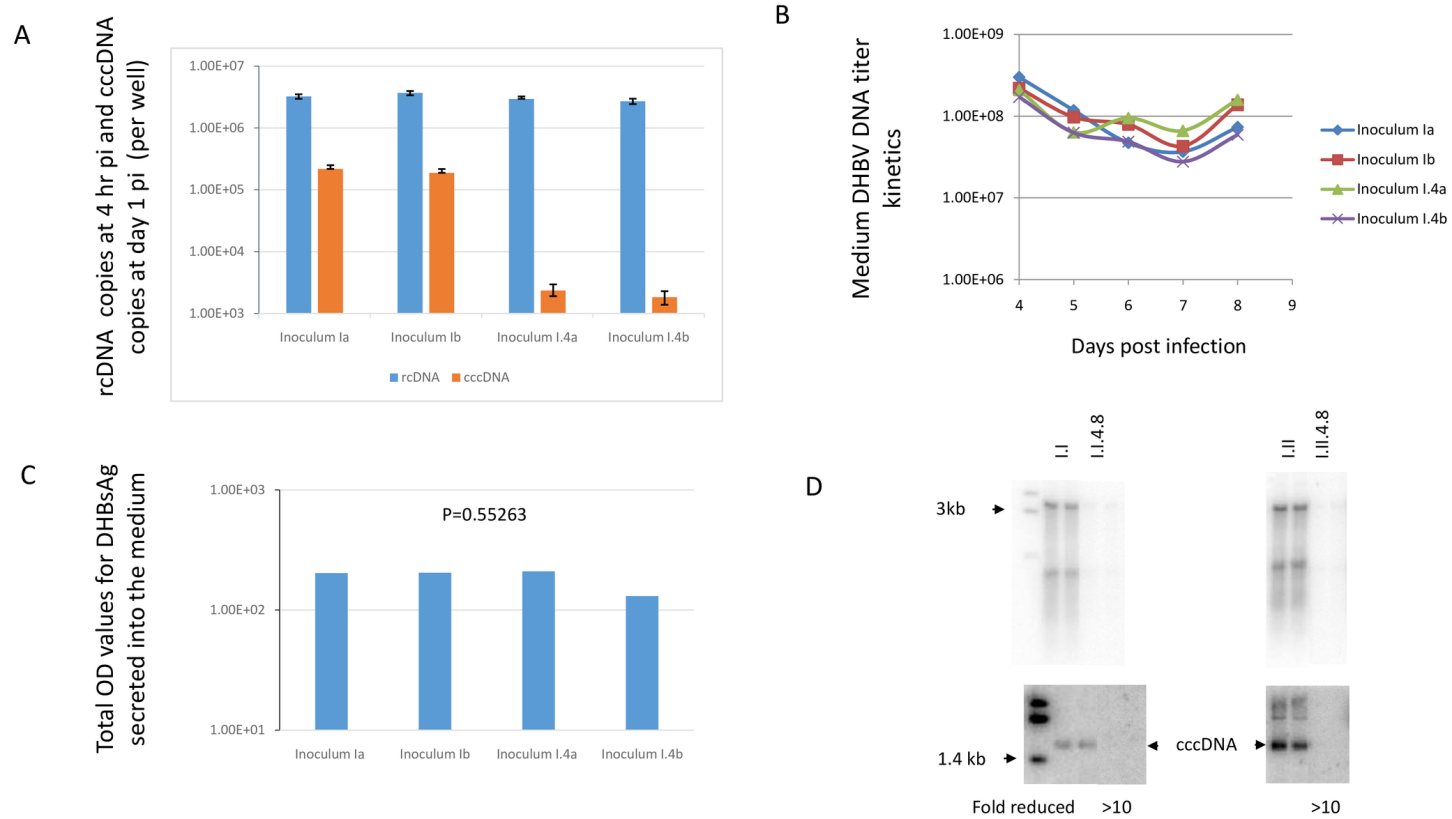
A significant reduction of cccDNA amplification was detected both at day 1 and day 8 post inoculation (pi). **A.** the intracellular rcDNA level reflecting the amount of viruses entered, was quantitatively determined by qPCR in the infected cells that were harvested at 4 hours pi. The cccDNA amplification was quantitatively determined in the cells at day 1 pi. One of six similar experiments is shown. Inoculum Ia and Ib stored at -20 C and Inoculum I.4a and I.4b stored at 4 C for 8 weeks. The error bars were plotted with SD from duplicate of the same samples. **B.** Similar DHBV DNA titers were detected in the medium between two inocula infected cells from day 4 to day 8 pi. The DHBV DNA copies detected at day 4 represented the sum accumulated between day 2 and 4 pi. One of three similar experiments is shown. The sample IDs were the same as 2A. **C**. similar amounts of DHBsAg were detected in the medium samples. The relative amount of DHBsAg in medium samples from day 4 to 8 was expressed as the total OD value (see methods). **D.** the Southern blots showed the clear differences in replicative intermediates and cccDNA level between two inocula infected cells harvested at day 8 pi (each sample tested in duplicate). The fold differences in cccDNA level between two inocula infected cells were calculated with Phosphorimager software (ImageQuant 5.2, GE). One of five similar experiments is shown. I.I and I.II: inocula stored at -20 C; I.I.4.8 and I.II.4.8: the same aliquots stored at 4 C for 8 weeks.

To further determine if the reduced accumulation of intracellular replicative intermediates resulted from reduced virus entry when treated serum inoculum was used, we directly compared the efficiency of viral entry following incubation with either the treated or standard serum inocula. Inoculated cells were treated with 0.25% trypsin for 15 min to remove non-specifically bound viruses from cells before harvest. The intracellular viral rcDNA was quantitatively determined by qPCR. As shown in Fig 2A, no significant difference in the amount of rcDNA was observed between two inocula infected cells. Next we investigated kinetics of DHBV DNA in culture supernatant and no substantially different patterns were noted between infected cultures using treated or standard inocula (Fig 2B). Note that the higher DHBV DNA level at day 4 medium in all samples reflected the accumulation of released virions from day 2 to day 4 since no medium was collected until day 4 after the inoculum was removed at day 1 postinfection (pi). We also concluded that about 4E6 residual inoculum signal after removal of the inoculum at day 1 pi did not have substantial impact on DHBV DNA copy numbers determined in day 4 or later medium (The standard inoculum size is 4E7 DHBV DNA for a 6cm dish cells with 4 ml medium, resulting in approximately 1E7/ml of DHBV DNA titer in the inoculum-containing medium. The inoculum medium was replaced with fresh one next day that removed about 90% inoculum virus signal. Average DHBV DNA copies in the day 4 medium were approximately 1E8 copies/ml, far greater than the 4E6 residual inoculum signal). There was no significant difference in the total OD value of detected DHBsAg between two inocula infected cultures (Fig 2C). These results demonstrate that the infection efficiency with the treated inoculum is comparable to the standard inoculum.

The DHBV cccDNA formation and amplification occur as early as day 1 post inoculation. For instance, we compared the cccDNA amplification efficiency in young ducklings and adolescent ducks after inoculation. Liver biopsies were performed at day 1 p.i. in 8 of 3-day old ducklings and 10 of 3-week old ducks. The cccDNA copies per cell were determined using the single nucleus based detection. We found average cccDNA and R.I. copies per cell at day 1 were 6.0±2.4 and 15.1±6.4 in 3-day old ducklings while the cccDNA and R.I. copies per cell were 1.3±0.7 and 7.7±6.9 in 3-week old ducks, respectively[38].

We further analyzed the early cccDNA amplification efficiency in the two infected cultures harvested at day 1 post inoculation. A significant difference in the cccDNA amplification as detected (Fig 2A), by qPCR was observed, suggesting that cccDNA amplification was negatively impacted in the cells infected with the treated inoculum. The successful detection of cccDNA at day 1 pi in infected PDH is consistent with the successful detection of cccDNA at day 1 pi in the experimentally infected ducks [38]. A significant reduction in both the cccDNA and replicative intermediates was observed in cells inoculated with treated inocula at day 8 pi with southern blots (Fig 2D). These results suggest that the reduced accumulation of replicative intermediates is accompanied by reduced cccDNA amplification in the treated inoculum infected cells.

### Different normal duck serum samples that were added to the treated serum infected cells resulted in different levels of restoration of cccDNA amplification

**Fig 3:**
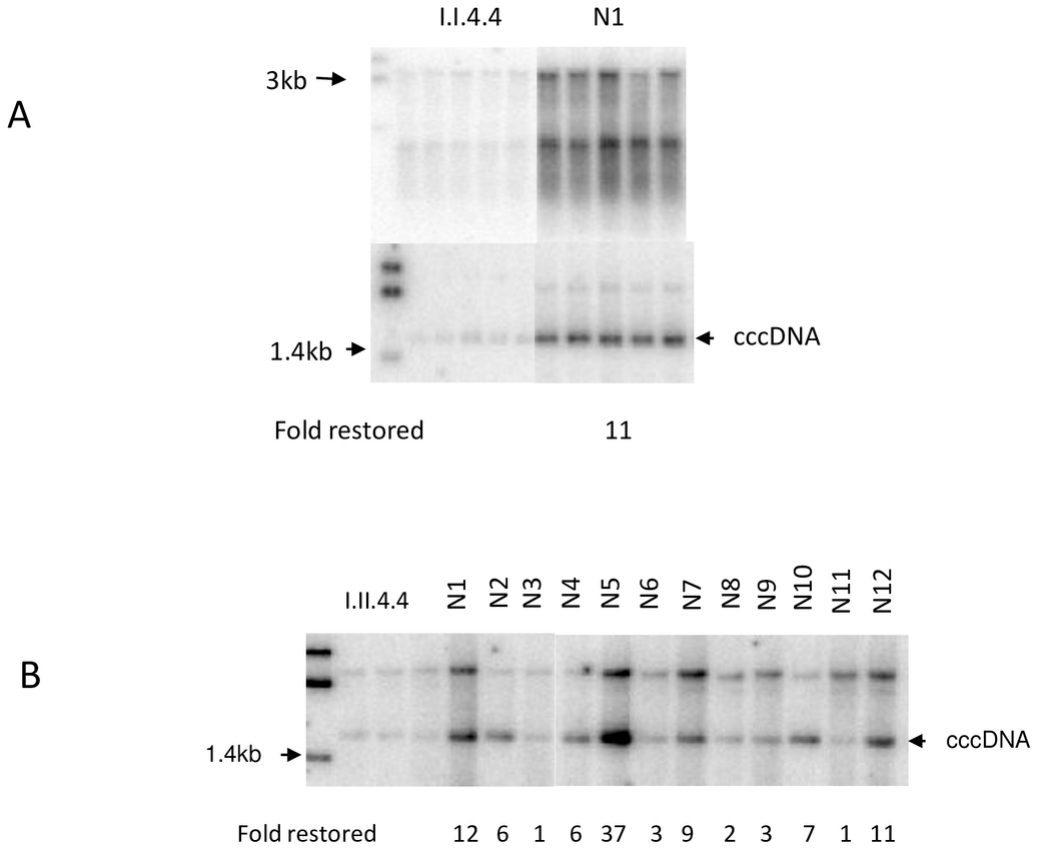
Restoration of cccDNA amplification by addition of 150 μl normal duck serum. **A**. Replicative intermediates and cccDNA were respectively detected in the cells added with 150 μl normal duck serum (N1) upon the inoculation with I.I.4.4 (4 C for 4 weeks) (each sample tested in quintuplicate). **B**. cccDNA detected in the I.II.4.4 (inoculum II stored at 4 C for 4 weeks) inoculated cells added with 150 μl normal duck serum collected from each of 12 animals (N1-N12). The restored fold was calculated based on the average (triplicate or quintuplicate) or the directly measured intensity (ImageQuant 5.2, GE) between samples with and without added normal serum.

As shown in Fig 1B, the addition of 150 μl normal duck serum to the treated serum infected cells restored the accumulation of the intracellular replicative intermediates and the cccDNA amplification (Fig 3A). A panel of 12 normal duck serum samples were tested for their capacity to restore cccDNA amplification. As shown in Fig 3B, the efficiency for restoring cccDNA amplification varied from different serum samples (1 to 37 fold).

### The efficiency of cccDNA amplification in WT infected culture was regulated through virion secretion efficiency

We inoculated PDH with either treated or standard serum inocula. The inoculum was replaced with the fresh medium at day 1 pi. The culture medium was daily changed and collected from day 4 to day 8. All cells were harvested at day 8 pi. The total rcDNA in the medium, the total rcDNA in the cells and total cccDNA were quantitatively analyzed by qPCR.

**Fig 4:**
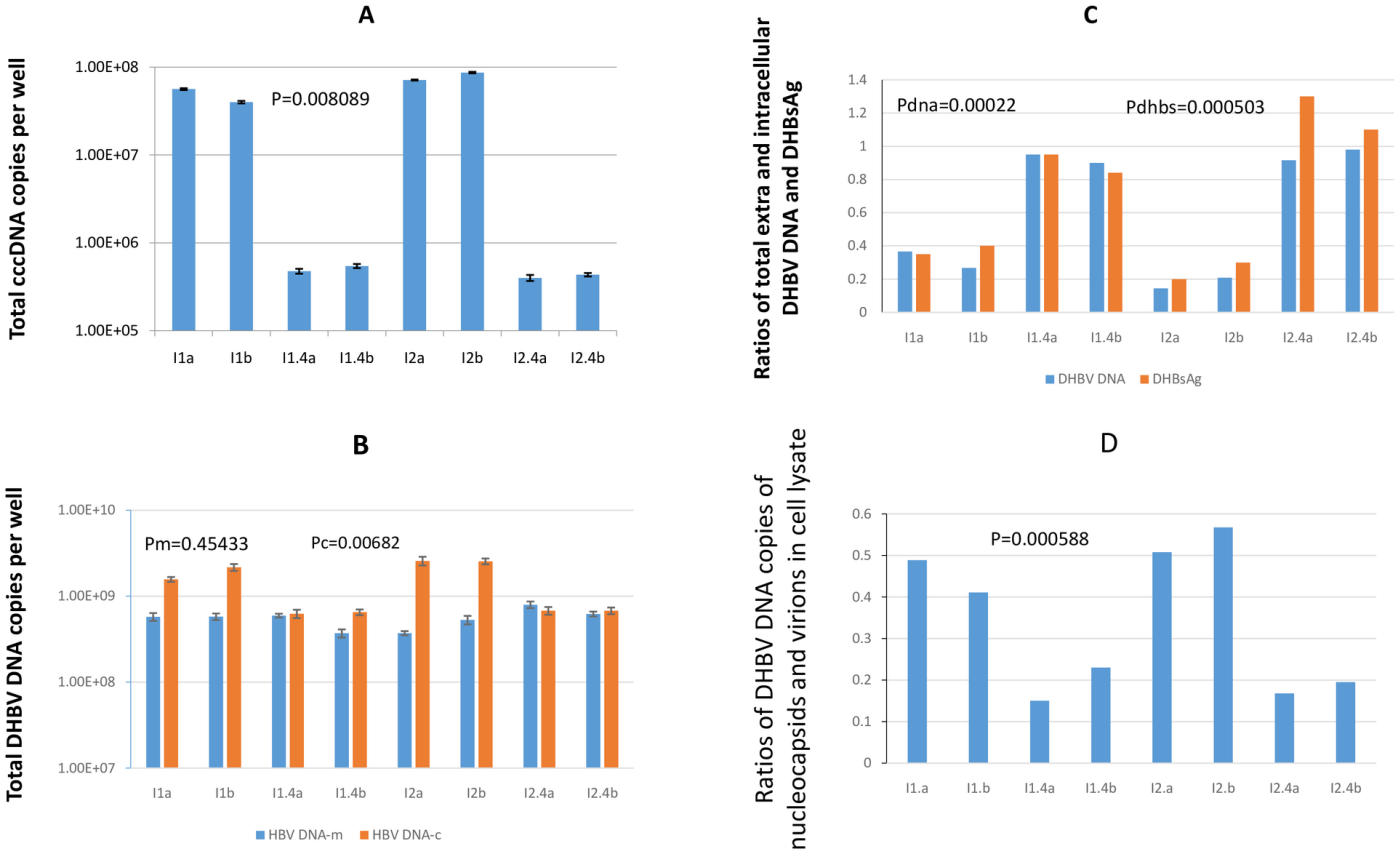
cccDNA amplification efficiency was regulated by virus secretion efficiency in DHBV WT infected primary hepatocytes. **A.** Approximate 80–100 fold differences in cccDNA level were detected in two inocula infected cells that were harvested at day 8 pi (duplicate for each sample tested). P value was calculated between two inocula: one consisting of I1a, I1b, I2a and I2b stored at -20 until inoculation; and the other consisting of I1.4a, I1.4b, I2.4a and I2.4b, the same aliquots to I1 and I2, but stored at 4 C for 8 weeks. The same calculations were applied to P values in **B, C** and **D**. **B**. Total extracellular DHBV DNA (DHBV DNA-m), and intracellular DHBV DNA (DHBV DNA-c) were quantitatively determined for each infected well. The total medium DHBV DNA was calculated as the sum of DHBV DNA in the medium from day 4 to 8 pi. Pm for medium DHBV DNA; Pc for cellular DHBV DNA. One of three similar DHBV infection experiments is shown. **C.** Ratios of total extracellular DHBV DNA and DHBsAg and intracellular DHBV DNA and DHBsAg between two inocula infected cells. Sample IDs were identical to those in Fig 4B. Pdna for extra and intracellular DHBV DNA and Pdhbs for extra and intracellular DHBsAg. One of three similar DHBV infection experiments is shown. **D**. Ratios of DHBV DNA copies detected in two captured fractions by respective rabbit anti-DHBV core protein and mouse anti-DHBsAg antibody. The same sample IDs were as listed in 4B. One of three similar DHBV infection experiments is shown.

As showed in Fig 4A, infection with the treated serum resulted in approximately 80-100-fold reduction in cccDNA amplification compared to infection with the standard serum, confirming different efficiencies of cccDNA amplification by the two serum inocula (P = 0.0081). We did not observe a significant difference in the amount of rcDNA in the medium (P = 0.45), however, we noted significant difference in the intracellular rcDNA level between the treated and standard (Fig 4B, P = 0.0068). When we analyzed the efficiency of virion secretion by comparing relative ratios between total extracellular rcDNA and intracellular viral DNA, we observed that the cells with higher cccDNA amplification efficiency (infected with standard serum) had lower secretion efficiency comparing the cells with lower cccDNA amplification efficiency (treated serum, Fig 4C, P = 0.0002). A similar pattern was detected when measuring the ratios of extracellular and intracellular DHBsAg (Fig 4C, P = 0.0005).

Finally, we determined relative ratios of DHBV DNA detected in the intracellular nucleocapsids and virions. A significantly higher rcDNA level was associated with the anti-DHBc-captured nucleocapsids in the cells infected with the standard serum and containing higher cccDNA amplification comparing that in the cells with lower cccDNA amplification (P = 0.0006), indicating that the reduced virus secretion resulted in a higher portion of nucleocapsids that were not enveloped (Fig 4D).

## Discussion

The purpose of this study was to investigate cccDNA amplification in a cell culture system, which resembles natural hepadnaviral infection in which the synthesis of envelope proteins was not experimentally ablated and the experimental manipulation was kept at minimum.

The previous studies identified the envelope proteins as a regulatory molecule for cccDNA amplification [12, 15–18] in the mutation based system. However, it remains unknown how cccDNA amplification is regulated during natural infection. In humans, chronic HBV infection produces and maintains a huge pool of envelope proteins, the majority of which are presented as HBsAg subviral particles that also contain both M and L proteins in addition to S major component. The ratio of HBsAg subviral particle to HBV virion is about 1000–10,000:1 [21] and a stable serum HBsAg level can be as high as 10,000 ng/ml [39]. The presence of high HBsAg level makes regulation of HBsAg level inefficient. It raises a question whether HBsAg or other viral molecules including virions are used to regulate cccDNA amplification in natural HBV infection. This study demonstrates that virion secretion efficiency, but not cellular envelope proteins level, is used to regulate cccDNA amplification efficiency in natural hepadnaviral infection.

In infected cells with higher cccDNA amplification, we found that the relative virus secretion efficiency was significantly lower, while the converse was true in infected cells with lower cccDNA amplification. The direct impact of changed virus secretion efficiency is exerted on the intracellular rcDNA level. Reduced virus secretion leads to increased rcDNA accumulation in the cells while increased virus secretion leads to reduced intracellular accumulation of rcDNA.

We detected a higher portion of free nucleocapsids that were captured by the anti-DHBc antibody in the cells with higher cccDNA amplification efficiency, suggesting more available nucleocapsids for the intracellular pathway once the secretion efficiency is down-regulated. Our results also suggest that the intracellular rcDNA level is an important factor determining the destination of the synthesized rcDNA and occupies the central position in regulating cccDNA amplification. The higher intracellular rcDNA level the more rcDNA molecules are available for cccDNA amplification. The role of the intracellular rcDNA level in regulating cccDNA level is consistent with the observed impact of nucleos(t)ide analogues (NA) on cccDNA level in treated patients. The NAs do not directly inhibit the cccDNA synthesis, but they do impact the cccDNA level through lowering intracellular rcDNA level in inhibiting DNA replication. Approximately 100 fold and 98% reduction of intracellular viral DNA in the NA-treated livers was detected, respectively, which was accompanied by approximately 100 fold and 84% reduction of cccDNA level, respectively [23, 40]. Another study showed that median intrahepatic total HBV DNA and cccDNA were decreased by 2.2 and 2.4log10 at the end of 48 weeks of antiviral therapy, respectively [24].

We also found that the intracellular DHBsAg accumulation was higher in cells with higher accumulation of rcDNA and cccDNA amplification, and cells with lower cccDNA amplification had lower rcDNA and DHBsAg accumulation (Fig 4C). This finding that is opposite to the inverse relationship between the envelope proteins and cccDNA amplification level observed in the mutation based system, suggests that the cellular envelope proteins concentration is not the direct target of regulation, but the virion level is in WT infected cells. Nonetheless, this study provides a new clue to studying cccDNA regulation in natural infection and a simple approach to prepare two DHBV serum inocula that are identical in all aspects except cccDNA amplification.

Initially, we thought that DHBV virions were damaged at 4 C storage. However, when a small volume (150 μl) of normal duck serum was added to the treated inoculum infected cells, both replicative intermediates and cccDNA were restored to the comparable levels detected in the standard inoculum infected cells. This observation led to the conclusion that the functionality of virions was not significantly compromised at 4C. A possible reason for reduced cccDNA amplification was that serum component(s) that may involve in down-regulating the efficiency of virion secretion, was damaged or degraded at 4C storage, because an inclusion of small volume of the normal duck serum can restore the cccDNA amplification efficiency.

Our data suggest that the virion and DHBsAg secretion efficiency from infected cells is subjected to regulation by an unknown serum factor, which may bind to the cellular membrane and negatively affect secretion of virions and subviral particles (Fig 4C) though the mechanism for such down-regulation is not known this time. The capacity to restore cccDNA amplification varies with different normal duck serum added (Fig 3B). Thus regulation of cccDNA amplification in WT infected cells involves both the host and viral molecules. The excessive accumulation of envelope proteins in the cells is reported in clinical HBV infection [41]. For instance, the Ground-Glass cells can be detected in the livers of patients with chronic HBV infection. The cytopathic appearance of Ground Glass cell results from excessive accumulation of viral envelope proteins[42], which can be caused by increased synthesis of envelope proteins, but not accompanied by corresponding increase of secretion efficiency or accompanied by reduced secretion efficiency. Either scenario implicates the involvement of host factors.

Understanding of regulation of cellular membrane secretion efficiency is not only important for HBV secretion and cccDNA amplification, but also is valuable for illustrating why biological molecules like fat or bile acids can be excessively accumulated in the hepatocytes, which cause fatty liver disease or cholestasis. The serum factors involved in regulating the virus secretion can be identified with proteomic analysis in the future. We believe that publication of this study will facilitate collective efforts in the field to identify this serum factor.

## Conclusions

In this study we report a simple procedure to prepare two identical duck hepatitis B virus inocula, but they possess 10-100-fold difference in cccDNA amplification in infected cell culture. We demonstrate that the infected cells with higher cccDNA amplification significantly reduce the virus secretion efficiency that results in higher accumulation of relaxed circular DNA (rcDNA) and DHBsAg in the cells. The infected cells with lower cccDNA amplification significantly increase the virus secretion efficiency that leads to lower intracellular rcDNA and DHBsAg accumulation. The regulation of cccDNA amplification in natural hepadnaviral infection bypasses direct regulation of the cellular envelope proteins concentration, instead it modulates virus secretion efficiency that ultimately impacts the intracellular rcDNA concentration, an important factor determining the destination of the synthesized rcDNA in infected cells.
